# Outlier detection in spatial error models using modified thresholding-based iterative procedure for outlier detection approach

**DOI:** 10.1186/s12874-024-02208-3

**Published:** 2024-04-15

**Authors:** Jiaxin Cai, Weiwei Hu, Yuhui Yang, Hong Yan, Fangyao Chen

**Affiliations:** 1 Department of Epidemiology and Biostatistics, School of Public Health, Xi’an Jiaotong University Health Science Center, No. 76, Yanta Xilu Road, Xi’an, 710061 Shaanxi China; 2 Key Laboratory for Disease Prevention and Control and Health Promotion of Shaanxi Province, Xi’an Jiaotong University, Xi’an, 710061 Shaanxi China; 3 Department of Radiology, First Affiliate Hospital of Xi’an Jiaotong University, Xi’an, 710061 Shaanxi China

**Keywords:** Outliers, Iterative procedure for outlier detection, Mean-shift outlier model, Spatial error model, Robust estimation

## Abstract

**Background:**

Outliers, data points that significantly deviate from the norm, can have a substantial impact on statistical inference and provide valuable insights in data analysis. Multiple methods have been developed for outlier detection, however, almost all available approaches fail to consider the spatial dependence and heterogeneity in spatial data. Spatial data has diverse formats and semantics, requiring specialized outlier detection methodology to handle these unique properties. For now, there is limited research exists on robust spatial outlier detection methods designed specifically under the spatial error model (SEM) structure.

**Method:**

We propose the Spatial-Θ-Iterative Procedure for Outlier Detection (Spatial-Θ-IPOD), which utilizes a mean-shift vector to identify outliers within the SEM. Our method enables an effective detection of spatial outliers while also providing robust coefficient estimates. To assess the performance of our approach, we conducted extensive simulations and applied it to a real-world empirical study using life expectancy data from multiple countries.

**Results:**

Simulation results showed that the masking and JD (Joint Detection) indicators of our Spatial-Θ-IPOD method outperformed several commonly used methods, even in high-dimensional scenarios, demonstrating stable performance. Conversely, the Θ-IPOD method proved to be ineffective in detecting outliers when spatial correlation was present. Moreover, our model successfully provided reliable coefficient estimation alongside outlier detection. The proposed method consistently outperformed other models (both robust and non-robust) in most cases. In the empirical study, our proposed model successfully detected outliers and provided valuable insights in the modeling process.

**Conclusions:**

Our proposed Spatial-Θ-IPOD offers an effective solution for detecting spatial outliers for SEM while providing robust coefficient estimates. Notably, our approach showcases its relative superiority even in the presence of high leverage points. By successfully identifying outliers, our method enhances the overall understanding of the data and provides valuable insights for further analysis.

**Supplementary Information:**

The online version contains supplementary material available at 10.1186/s12874-024-02208-3.

## Background

In general, an outlier refers to a data point that significantly deviates from the norm for a specific variable or population [1]. It is also characterized as an observation that is inconsistent with the remaining data [2]. Swersky et al. (2016) further defined an outlier as an observation that diverges to the extent of arousing suspicions [3]. Outliers are inevitable [4] and sometimes carry special information. While in practice, some outliers may simply be considered as “noise” or “dirty data”, more often than not, they have the potential to influence statistical inference and provide valuable insights within the dataset [5]. For instance, in a published breast cancer detection system, inliers may represent healthy patients, while outliers may indicate a higher probability of breast cancer [6]. As a result, incorrect or crude treatment of outliers often results in loss of information, inaccurate statistical inferences and biased estimates. Accurately identifying outliers, especially in the field of public health, is of significant importance for further analysis of outliers to provide additional insights in certain aspects. Therefore, the methodology for detecting outliers is an essential and urgent need in data analysis [5].

A dataset may contain multiple outliers, posing challenges in detecting and addressing the masking and swamping effects [7]. Various methods have been employed for multiple outlier detection, including the fully efficient one-step procedure (GY) proposed by Gervini and Yohai (2002) [8], the least trimmed squares (LTS) [9], and the MM-estimators [10]. Moreover, other methods have also been developed to tackle different aspects of outlier detection. For instance, Kong et al. (2018) proposed a method based on the squared loss of the mean-shift model with two penalty functions on the mean-shift vector and the parameter vector, achieving both high breakdown points and high efficiency [11]. Jiang et al. (2020) introduced the penalized weighted LAD-LASSO (PWLAD-LASSO) estimator, which combines robust estimation and outlier detection properties [12]. Among these methods, we noticed that the Θ-IPOD method proposed by She & Owen (2011) used a regression model with a mean shift parameter. They incorporated a soft-thresholding penalty and a hard-thresholding penalty, which effectively counter the masking effects [13].

However, in recent years, the presence of spatial heterogeneity in data has become increasingly common in various fields such as survey studies, surveillance efforts, and longitudinal studies, particularly in cancer-related research [14]. For instance, the Surveillance, Epidemiology, and End Results (SEER) Program, the China Health and Retirement Longitudinal Study (CHARLS), and the China Northwest Cohort (CNC) often involve the collection of data at small geographical levels (such as communities or counties), which are subsequently aggregated at larger levels. This introduces additional complexity to outlier detection tasks. The primary reason for this is that geographic data often exhibit spatial dependence [15]. Traditional methods for outlier detection fail to consider the spatial relationships among input variables, while spatial patterns often demonstrate spatial continuity and autocorrelation with neighboring samples. For instance, the Θ-IPOD method relies on a linear structure with a mean-shift vector. However, the existence of spatial dependence violates the assumptions of traditional ordinary least squares (OLS) estimation and can result in a decrease in the efficiency and increase in the bias of the OLS estimator for the regression model parameters [16]. There have been some approaches to spatial outlier exploration, however, due to the diverse formats and semantics of spatial data, there is still a urgent need for outlier detection methodology that can accommodate these unique properties especially spatial dependence and heterogeneity [17].

In the area of spatial analysis, one commonly used method is the spatial error model (SEM), which considers the covariance structure between error terms [18]. The SEM model is adept at effectively addressing challenges related to spatial correlation and heterogeneity. SEM has been successfully applied in various applications, providing valuable insights when the spatially autocorrelated error structure is well-defined [19]. Some robust spatial regression approaches have been proposed in recent years. José- Montero et al. (2012) introduced a model incorporating a global spatial trend within a Spatial Autoregressive (SAR) framework to address both large-scale spatial dependencies and local spatial autocorrelation. The utilization of penalized splines for model estimation was emphasized, leveraging their representation as mixed models [20]. Boente et al. (2012) presented a robust estimation framework encompassing parametric and nonparametric components within the context of a generalized partly linear single-index model [21]. Additionally, Yildirim et al. (2020) proposed a robust estimation approach utilizing robustified likelihood equations specifically tailored for SEM [22]. However, it is important to highlight that there is limited research available on robust spatial outlier detection specifically tailored to the SEM structure. These spatial robust estimation methods do not yield explicit results identifying which observations are outliers, which is not conducive to our further analysis of outliers.

Therefore, in this study, we propose a novel outlier detection method Spatial-Θ-IPOD for SEM-structure data. Considering the outstanding performance of the Θ-IPOD method in detecting outliers under normal circumstances, we have decided to extend its application to the structure of the SEM model to address the task of spatial outlier detection.

### The contributions of this paper are as follows:

(1) We proposed an extension of the IPOD method to incorporate the structure of the SEM model, calling Spatial-Θ-IPOD, enabling the detection of spatial outliers while effectively addressing the challenges posed by masking and swamping effects.

(2) In addition to outlier detection, our approach also provided robust estimates for the coefficients.

(3) We evaluated the effectiveness of the proposed algorithms for spatial outlier detection by applying them to the analysis of Life Expectancy (LE) data from multiple countries. We conduct a comprehensive analysis of the detected outliers, providing valuable insights and robust estimated results.

## Methods

### The Θ-IPOD method

The Θ-IPOD is based on the mean-shift model [13]:

1 $${\mathbf{y}} = {\mathbf{X\beta }} + {{\varvec{\upgamma}}} +\epsilon ,\quad \epsilon\sim {\mathcal{N}}\left( {0,\sigma^{2} {\mathbf{I}}} \right)$$

where ${\mathbf{X}} = [{\mathbf{x}}_{{\mathbf{1}}} ,...,{\mathbf{x}}_{{\mathbf{n}}} ]^{T} \in {\mathbb{R}}^{n \times p}$,${\mathbf{y}} = [y_{1} ,...,y_{n} ]^{T} \in {\mathbb{R}}^{n}$,${{\varvec{\upbeta}}} = [\beta_{1} ,...,\beta_{p} ]^{T} \in {\mathbb{R}}^{p}$,$\epsilon\in {\mathbb{R}}^{n}$ is a random error vector. ${{\varvec{\upgamma}}} = (\gamma_{1} ,...,\gamma_{n} )^{T} \in {\mathbb{R}}^{n}$ acts as a vector indicating the locations of outliers. If one *γ*_*i*_ does not equal 0, it means the corresponding observation is an outlier.

To deal with masking and swamping in the presence of multiple outliers mentioned before, *λ* is the regularization parameters, a general threshold function Θ was been used.$\Theta (t;\lambda )$ is an odd monotone unbounded shrinkage rule for *t*, at any *λ*, which satisfies:

1. $$\Theta ( - t;\lambda ) = - \Theta (t;\lambda )$$
2. $$\Theta (t;\lambda ) \le \Theta \left( {t^{\prime } ;\lambda } \right) \, for \, 0 \le t \le t^{\prime }$$
3. $$\mathop {\lim }\limits_{t \to \infty } \Theta (t;\lambda ) = \infty$$
4. $$0 \le \Theta (t;\lambda ) \le t \, for \, 0 \le t < \infty$$

In their study, they considered two version of threshold function Θ, which are:

2 $$\Theta_{{\text{soft }}} (x;\lambda ) \, = \left\{ {\begin{array}{*{20}l} {0,} \hfill & {{\text{ if }}|x| \le \lambda } \hfill \\ {x - {\text{sgn}} (x)\lambda ,} \hfill & {{\text{ if }}|x| > \lambda } \hfill \\ \end{array} } \right.$$

3 $$\Theta_{{\text{hard }}} (x;\lambda ) \, = \left\{ {\begin{array}{*{20}l} {0,} \hfill & {{\text{ if }}|x| \le \lambda } \hfill \\ {x,} \hfill & {{\text{ if }}|x| > \lambda } \hfill \\ \end{array} } \right.$$

For any threshold function Θ(·; λ), a penalty function $P_{\Theta } ( \cdot ;\lambda )$ with the smallest curvature corresponding can be found by following three-step construction,

- $$\Theta^{ - 1} (u;\lambda ) = \sup \{ t:\Theta (t;\lambda ) \le u\}$$
- $$s(u;\lambda ) = \Theta^{ - 1} (u;\lambda ) - u$$
- $$P(\theta ;\lambda ) = P_{\Theta } (\theta ;\lambda ) = \int_{0}^{|\theta |} s (u;\lambda ){\text{d}}u$$

The ultimate goal is to optimize the following formula to obtain the robust estimate of $({\hat{\mathbf{\beta }}},{\hat{\mathbf{\gamma }}})$ by iterative procedure.

4 $$f_{P} ({\mathbf{\beta ,\gamma }}) \equiv \frac{1}{2}||{\mathbf{y - X\beta - \gamma}}||_{2}^{2} + \sum\limits_{i = 1}^{n} P \left( {\gamma_{i} ;\lambda_{i} } \right)$$

The update of ${{\varvec{\upgamma}}}$ via ${{\varvec{\upgamma}}}^{(j + 1)} = \Theta \left( {{\mathbf{H\gamma }}^{(j)} + ({\mathbf{I}} - {\mathbf{H}}){\mathbf{y}};{{\varvec{\uplambda}}}} \right)$ at each iteration, where $\lambda_{i} = \lambda \sqrt {1 - h_{i} }$, the *HatMatrix* ${\mathbf{H}}$ can be defined as ${\mathbf{H}} = {\mathbf{H(X)}} = {\mathbf{X(X}}^{{\mathbf{T}}} {\mathbf{X)}}^{{{\mathbf{ - 1}}}} {\mathbf{X}}^{{\mathbf{T}}}$, $h_{i}$ donates the *i*th diagonal entry of ${\mathbf{H}}$.

About the choice of the regularization parameter, the *λ* can be chosen via BIC (Bayesian information criterion) [23, 24]. To be more specific, it can be chosen by a slight modification BIC. Given $\lambda$ and the corresponding estimate $\widehat{\gamma }(\lambda )$, let $nz(\lambda ) = \{ i:\widehat{\gamma }_{i} (\lambda ) \ne 0\}$,$\widehat{\gamma }_{nz}$ is an OLS estimate with one parameter per detected outlier, and the degrees of freedom are given by $DF(\lambda ) = \left| {nz(\lambda )} \right|$. The slight modification of BIC is as ${\text{BIC}}^{*} (\lambda ) = m\log ({\text{RSS}} /m) + k(\log (m) + 1)$,where $\mathbf{\overset{\frown}{y}}= {\mathbf{A\gamma }} + \epsilon^{\prime } ,\quad \epsilon^{\prime } \sim {\mathcal{N}}\left( {{\mathbf{0}},\sigma^{2} {\mathbf{I}}_{(n - p) \times (n - p)} } \right)$, $\mathbf{\overset{\frown}{y}}= {\mathbf{U}}_{{\mathbf{c}}}^{{\mathbf{T}}} {\mathbf{y}}$, ${\mathbf{A}}$ can be obtained by the spectral decomposition of *HatMatrix* ${\mathbf{H}}$, ${\mathbf{H = ADA}}^{{\mathbf{T}}}$, $m = n - p$,${\text{RSS}} = ||\mathbf{\overset{\frown}{y}}- {\mathbf{A}}\widehat{{{\varvec{\upgamma}}}}||_{2}^{2} = ({\mathbf{I}} - {\mathbf{H}})({\mathbf{y}} - \widehat{{{\varvec{\upgamma}}}})||_{2}^{2}$, and *k* = degrees of freedom + 1.

The selection range of $\lambda$ is decreasing from $||({\mathbf{I}} - {\mathbf{H}}){\mathbf{y}}/\sqrt {{\text{diag}} ({\mathbf{I}} - {\mathbf{H}})}||_{\infty }$ to 0, and select the $\lambda$ with the minimum ${\text{BIC}}^{*} (\lambda )$.

The detail algorithm is as follows:

**Figure:**
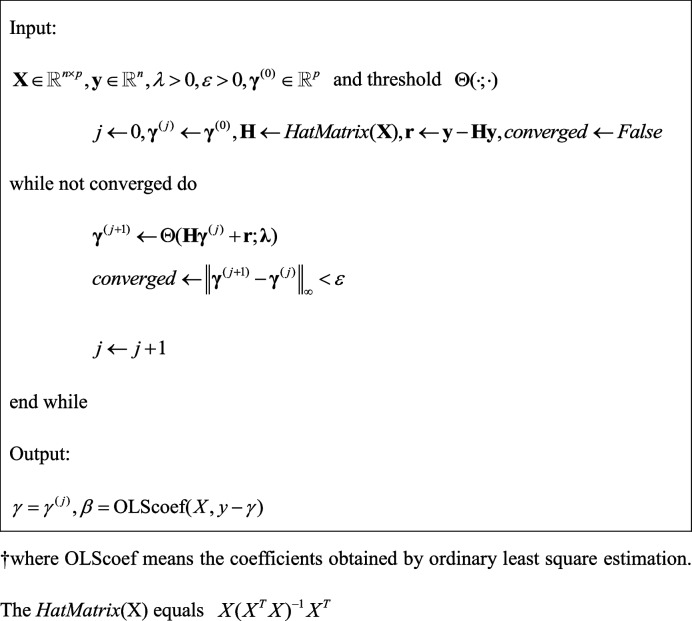
**Algorithm 1** Θ-IPOD

### Spatial error model

SEM has been extensively utilized in various fields such as econometrics, regional science, forest science, social science, and marketing research. More recently, it has also found applications in the field of public health [25]. SEM regression model involving the coefficient of spatial dependence or autocorrelation (*μ*) that captures the spatial dependence in the error terms, is presented as follows:

Normal SEM model can be described as

5 $${\mathbf{y}} = {\mathbf{X\beta }} + {{\varvec{\upxi}}},{{\varvec{\upxi}}} = \mu {\mathbf{W\xi }} +\epsilon ,\quad \epsilon\sim {\mathcal{N}}\left( {0,\sigma^{2} {\mathbf{I}}} \right)$$

where ${\mathbf{y}}$ contains an *n* × 1 vector of dependent variables and ${\mathbf{X}}$ represents an *n* × *p* matrix of independent variables. ${{\varvec{\upbeta}}}$ is a vector of *p* × 1 vector of regression parameter to be estimated of the model. *μ* is the spatial autoregressive parameter needed to be estimated. ${\mathbf{W}}$ is the row-standardized weight matrix, which is calculated based on the distance matrix indicating how locations are spatially interconnected. The lag-error term ${{\varvec{\upxi}}} = \mu {\mathbf{W\xi }} +\epsilon ,\quad \epsilon\sim {\mathcal{N}}\left( {0,\sigma^{2} {\mathbf{I}}} \right)$ effectively addresses spatial dependence within the error terms, thereby augmenting the conventional linear model. The Eq. (4) shows that the observations have a Gaussian distribution with ${\mathbf{y}}\sim {\mathcal{N}}({\mathbf{X\beta }},\sigma^{2} ({\mathbf{I}}_{n} - \mu {\mathbf{W}})^{ - 1} )$.

### Spatial-Θ-IPOD

As mentioned earlier, while Θ-IPOD demonstrates excellent performance under normal regression assumptions, it is observed that the error term deviates from the ordinary linear model. Consequently, Θ-IPOD may no longer be applicable in such cases.

To address this limitation, we propose a modified approach called Spatial-Θ-IPOD, which incorporates a mean shift vector *γ* into the SEM to identify outliers and obtain robust coefficient estimations. This modification enables the method to be suitable for the SEM data structure. The model is described as follows:

6 $${\mathbf{y}} = {\mathbf{X\beta }} + {{\varvec{\upgamma}}} + {{\varvec{\upxi}}},{{\varvec{\upxi}}} = \mu {\mathbf{W\xi }} + \epsilon,\quad \epsilon\sim {\mathcal{N}}\left( {0,\sigma^{2} {\mathbf{I}}} \right)$$

Motivated by Yildirim (2020) [22], one possible approach for estimating the regression coefficients of the SEM is the generalized least squares (GLS) method. This method is applicable when the spatial autoregressive parameter *μ* is known or has been previously estimated. Therefore, we generalize Eq. (5) as follows:

7 $${\tilde{\mathbf{Y}}} = {\mathbf{\tilde{X}\beta }} + {\tilde{\mathbf{\gamma }}} + \tau ,\quad \tau \sim {\mathcal{N}}\left( {0,\sigma^{2} {\mathbf{I}}} \right)$$

where $${\tilde{\mathbf{y}}} = ({\mathbf{I}}_{n} - \hat{\mu }{\mathbf{W}}){\mathbf{y}},{\tilde{\mathbf{X}}} = ({\mathbf{I}}_{n} - \hat{\mu }{\mathbf{W}}){\mathbf{X}},{\tilde{\mathbf{\gamma }}} = ({\mathbf{I}}_{n} - \hat{\mu }{\mathbf{W}}){{\varvec{\upgamma}}}$$.

Under this model setting, the optimization problem turns to

8 $$f_{P} ({{\varvec{\upbeta}}},{\tilde{\mathbf{\gamma }}}) \equiv \frac{1}{2}||{\tilde{\mathbf{y}}} - {\mathbf{\tilde{X}\beta }} - {\tilde{\mathbf{\gamma }}}||_{2}^{2} + \sum\limits_{i = 1}^{n} P \left( {\tilde{\gamma }_{i} ;\lambda_{i} } \right)$$

We utilize the iterative procedure to solve the optimization problem. Before that, if *μ* is known, it can directly be used for the optimization. If *μ* is unknown, it can be estimated previously by following method [22]:

- (i) Choose $$\psi$$ function
- (ii) Choose initial values $\beta$, $\mu$ via OLS (Ordinary least square) or GMM (Generalized Moment Model)
- (iii) Compute ${{\varvec{\upbeta}}}^{(i + 1)}$ from equation ${{\varvec{\upbeta}}}^{(i + 1)} = {{\varvec{\upbeta}}}^{(i)} + \left[ {I\left( {{{\varvec{\upbeta}}}^{(i)} } \right)} \right]^{ - 1} s_{\beta }^{(i)}$.
- (iv) Compute residuals with the estimated ${{\varvec{\upbeta}}}^{(i + 1)}$.
- (v) Compute $\mu^{(i + 1)}$ from equation $\mu^{(i + 1)} = \mu^{(i)} + [I(\mu^{(i)} )]^{ - 1} s_{\mu }^{(i)}$.
- (vi) Repeat steps iii-v until convergence for ${{\varvec{\upbeta}}}$ and $\mu$.

where ${\mathbf{r}} = \hat{\Omega }_{\lambda }^{ - 1/2} \frac{{({\mathbf{y}} - {\mathbf{X\beta }})}}{{\hat{\sigma }}}$,$\Omega_{\lambda } = \left( {{\mathbf{I}}_{n} - \lambda {\mathbf{W}}} \right)^{ - 1} \left( {{\mathbf{I}}_{n} - \lambda {\mathbf{W}}^{\prime } } \right)^{ - 1}$,$K = \int_{ - \infty }^{\infty } {\psi^{2} } (r)f(r)dr$, $\psi ( \cdot )$ is the influence function can be chosen, containing Cauchy function, Insha function, etc. The observed information matrix $I( \cdot )$ can be obtained as minus the expected value of the second derivatives of the robust log-likelihood functions. The score functions are $s_{\beta } = \, \frac{{\hat{\sigma }}}{{\sigma^{2} }}{\mathbf{X}}^{\prime } \left( {{\mathbf{I}}_{n} - \mu {\mathbf{W}}} \right)^{2} \left( {{\mathbf{I}}_{n} - \hat{\mu }{\mathbf{W}}} \right)^{ - 1} \psi (r) = 0$ and $s_{\mu } = - K{\text{tr}} \left( {\left( {{\mathbf{I}}_{n} - \mu {\mathbf{W}}} \right)^{ - 1} {\mathbf{W}}} \right) + \frac{{\hat{\sigma }^{2} }}{{\sigma^{2} }}\psi (r)^{\prime } \left( {{\mathbf{I}}_{n} - \hat{\mu }{\mathbf{W}}} \right)^{ - 1} \left( {{\mathbf{I}}_{n} - \mu {\mathbf{W}}} \right) \times {\mathbf{W}}\left( {{\mathbf{I}}_{n} - \hat{\mu }{\mathbf{W}}} \right)^{ - 1} \psi (r) = 0$.

The Spatial-Θ-IPOD algorithm is listed as follows:

**Figure:**
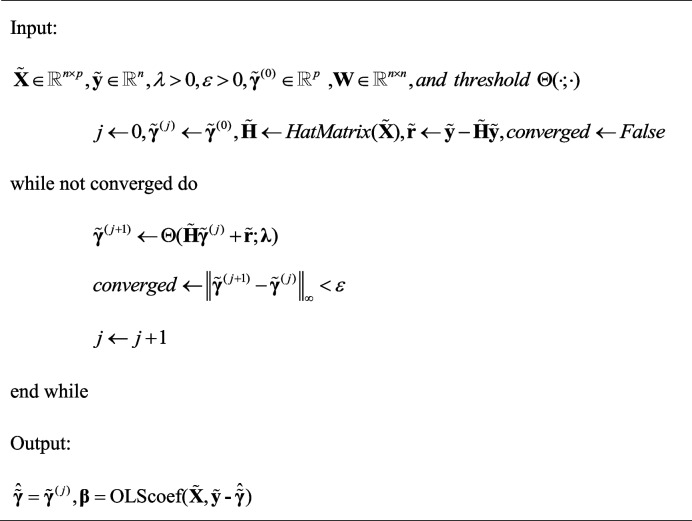
**Algorithm 2** Spatial-Θ-IPOD

Similar with IPOD, the regularization parameter of our proposed Spatial-Θ-IPOD is tuned in a data-dependent way by a slight modification of BIC, with decreasing $\lambda$ from $||({\mathbf{I}} - {\tilde{\mathbf{H}}}){\tilde{\mathbf{y}}}/\sqrt {{\text{diag}} ({\mathbf{I}} - {\tilde{\mathbf{H}}})}||_{\infty }$ to 0.

## Simulation study

### Simulation design

We carried out simulation experiments to test the performance of the Spatial-Θ-IPOD. It is well known that the presence of leverage points can cause failure in outlier detection methods. To be more specific, a data point whose x-value (independent) is unusual, y-value follows the predicted regression line though. Thus, we considered different combinations of dimensions, outlier quantities, and leverage values.

The observations were generated according to

9 $${\mathbf{y}} = {\mathbf{X\beta }} + {{\varvec{\upgamma}}} + {{\varvec{\upxi}}},{{\varvec{\upxi}}} = \mu {\mathbf{W\xi }} + \epsilon,\quad \epsilon\sim {\mathcal{N}}\left( {0,\sigma^{2} {\mathbf{I}}} \right)$$

The predictor matrix ${\mathbf{X}}$ is constructed as follows. Firstly, let ${\mathbf{X}} = {\mathbf{U\Sigma }}^{1/2}$, where $U_{ij} \mathop \sim \limits^{iid} U( - 5,5)$ and $\Sigma_{ij} = \rho^{{1_{i \ne j} }}$ with *ρ* = 0.5. The dimension of ${\mathbf{X}}$ is set $p \in \{ 15,50\}$, *n* = 500. Next, we modify the first *O* rows to represent leverage points, which are given by $L \cdot [1,...,1]$. We consider six cases, involving variations of $L \in \{ 15,20\}$ and $O \in \{ 10,20,50\}$. Additionally, three more cases involve additive outliers at *O* points that are not leverage points, meaning that no rows of ${\mathbf{X}}$ are changed. The *β* vector is set as [1,…,1]_*p*_. The shift vector is generated by ${{\varvec{\upgamma}}} = (\{ 8\}^{O} ,\{ 0\}^{n - O} )$. In order to add spatial heterogeneity, we incorporate a spatial error term ${{\varvec{\upxi}}}$ into the model. The generation of the spatial error term ${{\varvec{\upxi}}}$ is constructed as follows, with *λ* set to 0.7.

The spatial contiguity matrix ${\mathbf{W}} = ({\mathbf{W}}_{ij} )$ can be generated based on $$w_{ij} = \left\{ \begin{gathered} r^{|i - j|} ,i \ne j \hfill \\ 0,i = j \hfill \\ \end{gathered} \right.$$, where *r* = 0.5. Here, we assume that these observations are arranged in a linear sequence. Generally, it can be considered as a graph structure. The $\left| {i - j} \right|$ donates the graph distance between observation *i* and *j*. The *σ*^*2*^ is set 0.2.

### Our simulation experiments mainly contain two aspects:

The first part of our simulation experiments focuses on comparing the outlier detection performance of seven different methods: Spatial-hard-IPOD, Spatial-soft-IPOD, hard-IPOD, soft-IPOD, MM-estimator, fully efficient one-step procedure proposed by Gervini and Yohai (donoted by GY), and the least trimmed squares (LTS). These methods are implemented in the robust package (R version 4.1.2) and available in the S–PLUS Robust library. To ensure a fair comparison with Θ-IPOD, we evaluate their performance based on three benchmark measures: the mean masking probability (M), the mean swamping probability (S), and the joint outlier detection rate (JD).

The mean masking probability (M) represents the fraction of true outliers that go undetected. The mean swamping probability (S) indicates the fraction of non-outliers that are incorrectly labeled as outliers. The JD is the joint outlier detection rate, which measures the fraction of simulations with no masking (false negatives). In outlier detection, masking is considered more serious than swamping as it can lead to significant distortions. Swamping, on the other hand, typically results in a loss of efficiency. Ideally, we aim for M to be close to 0, S to be close to 0, and JD to be close to 100%. The joint outlier detection rate (JD) is particularly important for easier problems, while the mean masking probability (M) is more relevant for harder problems.

In the second part of our experiments, we compare the Mean Squared Error (MSE) of the estimated parameter *β* among 13 methods. These include the seven outlier detection methods mentioned earlier, as well as several robust methods for spatial estimation regression such as RoMLE (Robust estimation approach for spatial error model), including (RoMLE_Cauchy, RoMLE_Welsch, RoMLE_Insha, and RoMLE_Logistic). Because the RoMLE for SEM has smaller mean squared errors and exhibits more robust empirical influence function than the classical methods, when there are outliers in the dataset, we also conclude in our comparison. The difference between the four RoMLE method is that they choose different *ψ* function. The *ψ* function is introduced in Method section. Additionally, we consider non-robust methods, such as MLE (Maximum Likelihood Estimation) and GMM (Generalized Moments Method).

All calculations were performed in R. The code and scripts reproducing the examples in this simulation study are publicly available online at GitHub (https://github.com/Justin0607/spatialoutlierdetection).

## Simulation results

**Fig. 1:**
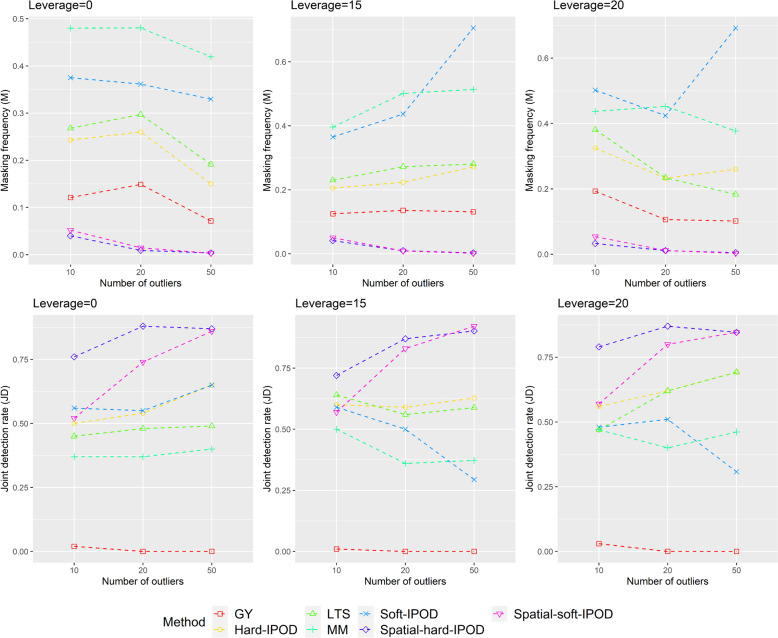
Masking (M) and joint detection (JD) when *p* = 15

**Fig. 2:**
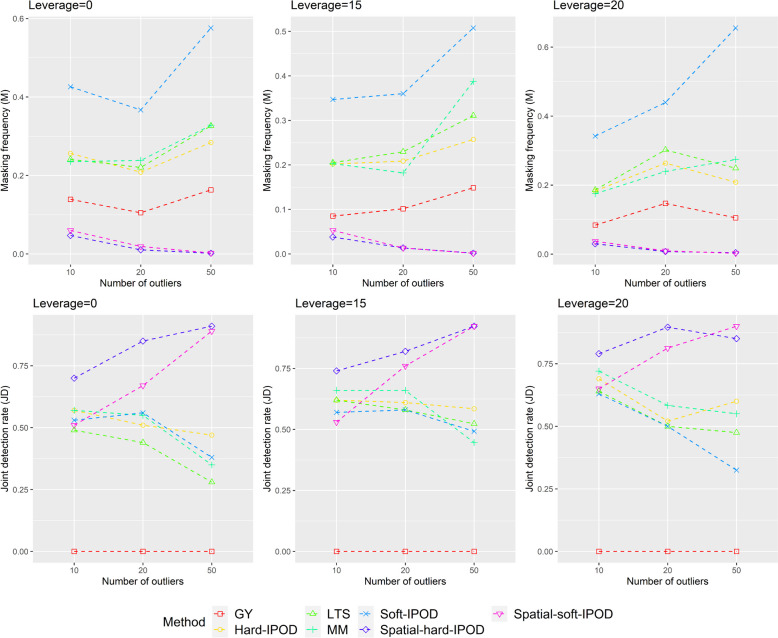
Masking (M) and joint detection (JD) when *p* = 50

Tables 1 and 2 present the outlier identification performances of seven models in various simulation scenarios. Figs. 1 and 2 illustrate the results of Masking and JD for *p* = 15 and 50 respectively. While our main objective is to identify outliers, our proposed Spatial-Θ-IPOD model also provides a robust coefficient estimate $\hat{\beta }$.

**Table 1:** Outlier identification results on simulated data with *p* = 15

	Outlier = 50			Outlier = 20			Outliers = 10		
	M	S	JD	M	S	JD	M	S	JD
No leverage									
Spatial-hard-IPOD	0.0036	0.0533	87	0.0090	0.0484	88	0.0400	0.0459	76
Spatial-soft-IPOD	0.0030	0.0518	86	0.0145	0.0195	74	0.0520	0.0129	52
Hard-IPOD	0.1500	0.0038	65	0.2600	0.0054	54	0.2430	0.0132	50
Soft-IPOD	0.3296	0.0072	65	0.3615	0.0016	55	0.3750	0.0004	56
MM	0.4192	0.0110	40	0.4805	0.0188	37	0.4800	0.0335	37
LTS	0.1914	0.0076	49	0.2970	0.0148	48	0.2680	0.0236	45
GY	0.0714	0.0680	0	0.1490	0.1120	0	0.1210	0.1221	2
Leverage = 15									
Spatial-hard-IPOD	0.0027	0.0505	90	0.0095	0.0439	87	0.0410	0.0445	72
Spatial-soft-IPOD	0.0016	0.0550	92	0.0090	0.0258	83	0.0500	0.0123	57
Hard-IPOD	0.2714	0.0041	63	0.2235	0.0058	59	0.2050	0.0058	60
Soft-IPOD	0.7059	0.0124	29	0.4365	0.0024	50	0.3650	0.0010	59
MM	0.5137	0.0117	37	0.5010	0.0259	36	0.3960	0.0358	50
LTS	0.2804	0.0108	59	0.2720	0.0162	56	0.2300	0.0220	64
GY	0.1310	0.0740	0	0.1355	0.1057	0	0.1250	0.1234	1
Leverage = 20									
Spatial-hard-IPOD	0.0046	0.0487	85	0.0110	0.0437	87	0.0330	0.0457	79
Spatial-soft-IPOD	0.0031	0.0542	85	0.0115	0.0238	80	0.0540	0.0127	57
Hard-IPOD	0.2600	0.0044	69	0.2325	0.0074	62	0.3250	0.0021	56
Soft-IPOD	0.6923	0.0207	31	0.4240	0.0032	51	0.5020	0.0008	48
MM	0.3769	0.0166	46	0.4525	0.0215	40	0.4370	0.0384	47
LTS	0.1831	0.0055	69	0.2340	0.0169	62	0.3810	0.0174	47
GY	0.1015	0.0699	0	0.1060	0.1138	88	0.1930	0.1213	3

Seven methods are compared: Our proposed hard-IPOD, our proposed hard-IPOD, hard-IPOD, soft-IPOD, MM-estimator, LTS and Gervini–Yohai’s fully efficient one-step procedure

**Table 2:** Outlier identification results on simulated data with *p* = 50

	Outlier = 50			Outlier = 20			Outliers = 10		
	M	S	JD	M	S	JD	M	S	JD
No leverage									
Spatial-hard-IPOD	0.0018	0.0564	91	0.0105	0.0518	85	0.0470	0.0420	70
Spatial-soft-IPOD	0.0024	0.0502	89	0.0190	0.0189	67	0.0600	0.0130	51
Hard-IPOD	0.2840	0.0097	47	0.2085	0.0034	51	0.2560	0.0060	57
Soft-IPOD	0.5760	0.0060	38	0.3670	0.0025	56	0.4260	0.0009	53
MM	0.3280	0.0976	35	0.2385	0.1201	55	0.2350	0.1421	57
LTS	0.3264	0.0312	28	0.2205	0.0462	44	0.2410	0.0493	49
GY	0.1632	0.1456	0	0.1050	0.1866	0	0.1390	0.2025	0
Leverage = 15									
Spatial-hard-IPOD	0.0018	0.0542	92	0.0135	0.0452	82	0.0380	0.0412	74
Spatial-soft-IPOD	0.0015	0.0534	92	0.0135	0.0221	76	0.0530	0.0129	53
Hard-IPOD	0.2572	0.0191	58	0.2085	0.0014	61	0.2020	0.0030	62
Soft-IPOD	0.5077	0.0097	49	0.3600	0.0026	58	0.3470	0.0006	57
MM	0.3877	0.0625	45	0.1820	0.1329	66	0.2030	0.1419	66
LTS	0.3108	0.0305	52	0.2295	0.0416	58	0.2050	0.0493	62
GY	0.1486	0.1431	0	0.1015	0.1853	0	0.0850	0.1992	0
Leverage = 20									
Spatial-hard-IPOD	0.0040	0.0513	85	0.0073	0.0438	90	0.0300	0.0440	79
Spatial-soft-IPOD	0.0025	0.0518	90	0.0094	0.0281	81	0.0370	0.0133	65
Hard-IPOD	0.2085	0.0026	60	0.2635	0.0067	52	0.1830	0.0028	69
Soft-IPOD	0.6555	0.0071	33	0.4396	0.0040	50	0.3420	0.0011	63
MM	0.2740	0.0860	55	0.2396	0.1254	58	0.1750	0.1426	72
LTS	0.2490	0.0261	48	0.3021	0.0452	50	0.1860	0.0500	64
GY	0.1050	0.1386	0	0.1469	0.1878	0	0.0840	0.1997	0

Seven methods are compared: Our proposed hard-IPOD, our proposed soft-IPOD, hard-IPOD, soft-IPOD, MM-estimator, LTS and Gervini–Yohai’s fully efficient one-step procedure

**Fig. 3:**
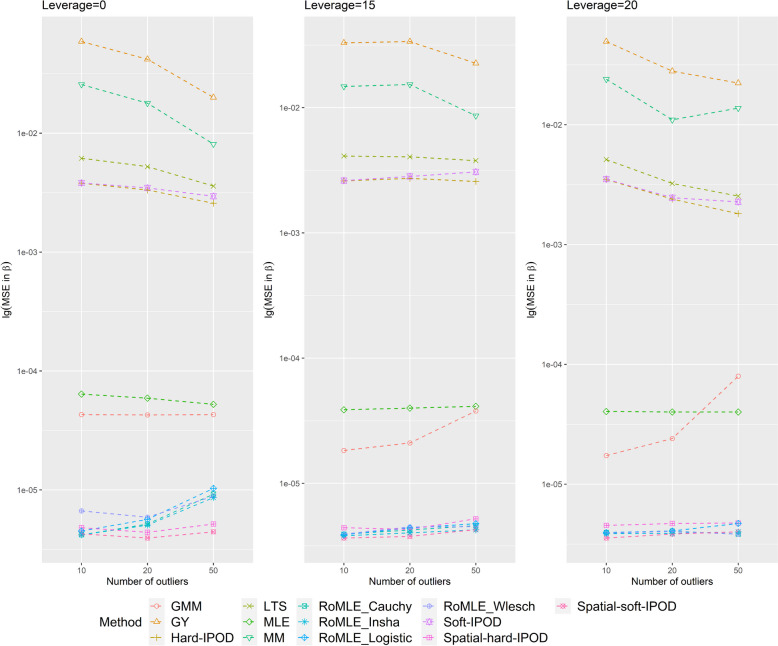
Coefficient estimation errors when *p* = 15

**Fig. 4:**
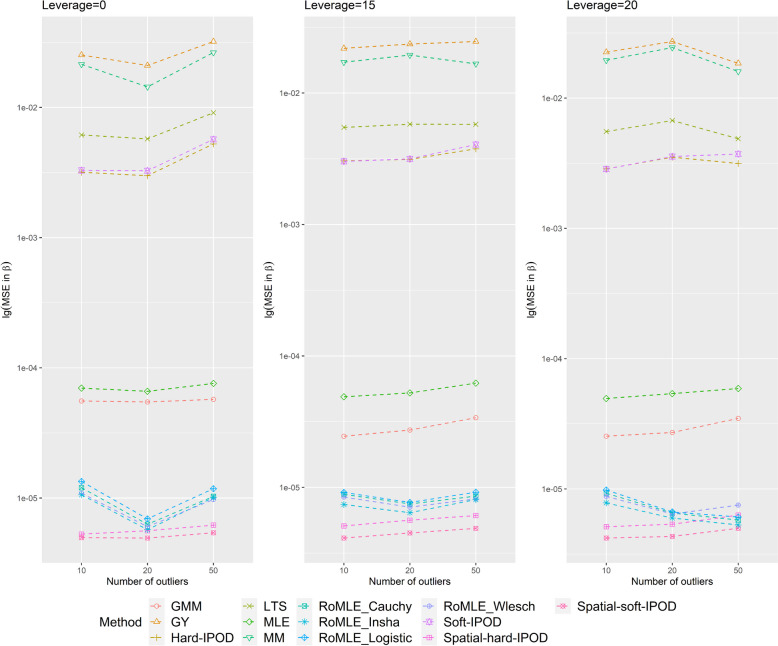
Coefficient estimation errors when *p* = 50

The MSE in *β* for *p* equals 15 and 50 can be found in Tables 3 and 4 respectively, with corresponding trends shown in Figs. 3 and 4. Because our model significantly outperforms other models, even by several orders of magnitude, we have applied a logarithmic transformation to the MSE for ease of visualization and to better illustrate the trend.

**Table 3:** MSE of beta (*p* = 15)

	Outlier = 50	Outlier = 20	Outlier = 10
No leverage			
Spatial-hard-IPOD	5.17E-06	4.40E-06	4.81E-06
Spatial-soft-IPOD	4.44E-06	3.94E-06	4.27E-06
Hard-IPOD	2.57E-03	3.33E-03	3.79E-03
Soft-IPOD	2.95E-03	3.46E-03	3.82E-03
RoMLE_Cauchy	9.21E-06	5.17E-06	4.19E-06
RoMLE_Welsch	8.85E-06	5.89E-06	6.66E-06
RoMLE_Insha	8.61E-06	5.05E-06	4.19E-06
RoMLE_Logistic	1.03E-05	5.66E-06	4.51E-06
MM	8.09E-03	1.79E-02	2.57E-02
LTS	3.59E-03	5.23E-03	6.15E-03
GY	2.00E-02	4.18E-02	5.90E-02
MLE	5.24E-05	5.91E-05	6.39E-05
GMM	4.30E-05	4.27E-05	4.30E-05
Leverage = 15			
Spatial-hard-IPOD	5.21E-06	4.29E-06	4.42E-06
Spatial-soft-IPOD	4.27E-06	3.77E-06	3.66E-06
Hard-IPOD	2.57E-03	2.73E-03	2.60E-03
Soft-IPOD	3.07E-03	2.82E-03	2.61E-03
RoMLE_Cauchy	4.59E-06	4.24E-06	3.93E-06
RoMLE_Welsch	4.51E-06	4.49E-06	3.93E-06
RoMLE_Insha	4.25E-06	4.03E-06	3.81E-06
RoMLE_Logistic	4.76E-06	4.40E-06	3.89E-06
MM	8.61E-03	1.53E-02	1.47E-02
LTS	3.78E-03	4.05E-03	4.11E-03
GY	2.26E-02	3.37E-02	3.29E-02
MLE	4.12E-05	3.99E-05	3.87E-05
GMM	3.78E-05	2.10E-05	1.83E-05
Leverage = 20			
Spatial-hard-IPOD	4.74E-06	4.69E-06	4.52E-06
Spatial-soft-IPOD	4.00E-06	3.83E-06	3.55E-06
Hard-IPOD	1.81E-03	2.39E-03	3.49E-03
Soft-IPOD	2.27E-03	2.46E-03	3.51E-03
RoMLE_Cauchy	3.83E-06	4.05E-06	3.95E-06
RoMLE_Welsch	3.82E-06	4.03E-06	3.91E-06
RoMLE_Insha	3.99E-06	3.88E-06	3.86E-06
RoMLE_Logistic	4.70E-06	4.06E-06	3.94E-06
MM	1.38E-02	1.10E-02	2.40E-02
LTS	2.54E-03	3.24E-03	5.12E-03
GY	2.23E-02	2.80E-02	4.95E-02
MLE	4.00E-05	4.00E-05	4.04E-05
GM	7.96E-05	2.40E-05	1.73E-05

Thirteen methods are compared: Spatial-hard-IPOD, Spatial-soft-IPOD, hard-IPOD, soft-IPOD, RoMLE_Cauchy, RoMLE_Welsch, RoMLE_Insha, RoMLE_Logistic, MM-estimator, LTS, Gervini–Yohai’s fully efficient one-step procedure, Maximum likelihood estimation (MLE) and Generalized moments method (GMM)

**Table 4:** MSE of beta (*p* = 50)

	Outlier = 50	Outlier = 20	Outlier = 10
No leverage			
Spatial-hard-IPOD	6.19E-06	5.61E-06	5.28E-06
Spatial-soft-IPOD	5.41E-06	4.93E-06	4.96E-06
Hard-IPOD	5.25E-03	2.99E-03	3.18E-03
Soft-IPOD	5.69E-03	3.27E-03	3.28E-03
RoMLE_Cauchy	1.03E-05	6.29E-06	1.20E-05
RoMLE_Welsch	9.74E-06	6.03E-06	1.09E-05
RoMLE_Insha	1.01E-05	5.71E-06	1.06E-05
RoMLE_Logistic	1.18E-05	6.92E-06	1.34E-05
MM	2.64E-02	1.44E-02	2.14E-02
LTS	9.10E-03	5.74E-03	6.14E-03
GY	3.21E-02	2.10E-02	2.53E-02
MLE	7.59E-05	6.60E-05	6.99E-05
GMM	5.73E-05	5.47E-05	5.56E-05
Leverage = 15			
Spatial-hard-IPOD	6.10E-06	5.63E-06	5.09E-06
Spatial-soft-IPOD	4.88E-06	4.50E-06	4.12E-06
Hard-IPOD	3.76E-03	3.13E-03	3.05E-03
Soft-IPOD	4.05E-03	3.15E-03	3.02E-03
RoMLE_Cauchy	8.58E-06	7.49E-06	8.86E-06
RoMLE_Welsch	8.17E-06	7.08E-06	8.43E-06
RoMLE_Insha	8.08E-06	6.42E-06	7.41E-06
RoMLE_Logistic	9.19E-06	7.68E-06	9.15E-06
MM	1.67E-02	1.94E-02	1.71E-02
LTS	5.78E-03	5.79E-03	5.47E-03
GY	2.46E-02	2.36E-02	2.19E-02
MLE	6.21E-05	5.23E-05	4.89E-05
GMM	3.39E-05	2.73E-05	2.45E-05
Leverage = 20			
Spatial-hard-IPOD	6.29E-06	5.36E-06	5.13E-06
Spatial-soft-IPOD	4.99E-06	4.32E-06	4.20E-06
Hard-IPOD	3.15E-03	3.51E-03	2.86E-03
Soft-IPOD	3.72E-03	3.56E-03	2.85E-03
RoMLE_Cauchy	5.77E-06	6.53E-06	9.26E-06
RoMLE_Welsch	7.52E-06	6.42E-06	8.78E-06
RoMLE_Insha	5.29E-06	5.98E-06	7.82E-06
RoMLE_Logistic	6.09E-06	6.66E-06	9.81E-06
MM	1.60E-02	2.44E-02	1.95E-02
LTS	4.88E-03	6.72E-03	5.53E-03
GY	1.85E-02	2.72E-02	2.25E-02
MLE	5.90E-05	5.39E-05	4.94E-05
GMM	3.48E-05	2.71E-05	2.54E-05

Thirteen methods are compared: Our proposed hard-IPOD, our proposed hard-IPOD, hard-IPOD, soft-IPOD, RoMLE_Cauchy, RoMLE_Welsch, RoMLE_Insha, RoMLE_Logistic, MM-estimator, LTS, Gervini–Yohai’s fully efficient one-step procedure, Maximum likelihood estimation (MLE) and Generalized moments method (GMM)

In terms of masking, our proposed model consistently outperforms the other models across all simulation scenarios when *p* equals 15. We also notice that both our Spatial-hard-IPOD and Spatial-soft-IPOD models exhibit similar performance (Tables 1 and  2, Figs. 1 and  2).

Additionally, we compare our models with three standard methods (MM, GY, and LTS) from the SPLUS Robust library. Among these, the GY-estimator ranks second in terms of performance. However, the MM-estimator, despite its popularity in robust analysis, and Spatial-soft-IPOD show relatively weaker performance. When *p* equals 50, the overall results remain largely consistent, with a slight improvement in MM's performance, although it still falls in the middle when compared to other models (Tables 1 and  2, Figs. 1 and  2).

In terms of the JD indicator, when *p* equals 15, our proposed model consistently outperforms the other models in most scenarios, except for one scenario with a small number of outliers and no leverage. In this particular scenario, the Spatial-soft-IPOD model falls slightly behind the soft-IPOD, but the Spatial-hard-IPOD still remains the top-performing model among all. In contrast, the performance of the hard-IPOD, soft-IPOD, MM, and LTS models is not as satisfactory. Notably, the GY-estimator performs poorly across all cases, indicating limited effectiveness in outlier detection even with a large number of simulations. When *p* = 50, we find that the performance of our proposed method is not significantly affected, as the JD indicators continue to remain at a high level (Tables 1 and  2, Figs. 1 and  2).

Regarding swamping, it is worth mentioning that although our proposed Spatial-Θ-IPOD model excels in masking, it shows slightly weaker performance in terms of swamping. However, this trade-off is acceptable, as masking poses a greater risk and harm.

Overall, the soft-IPOD, hard-IPOD, MM, LTS, and GY models demonstrate high masking probabilities and low joint detection rates, particularly when the dimensionality (*p*) is high. However, our proposed Spatial-Θ-IPOD method surpasses all of these models in terms of both masking probability and joint detection rate.

We also present the MSE of $\widehat{\beta }$. As depicted in Table 3, when *p* equals 15, it is evident that our method significantly outperforms other methods in most cases. The hard-IPOD, soft-IPOD, MM, LTS, and GY models exhibit considerably poorer performance, with a magnitude difference that is much larger compared to other models. The MLE and GMM models demonstrate better performance than the aforementioned five methods but still have room for improvement. Among all the models, the four RoMLE models are the closest to our MSE, but generally, our method still yields lower MSE, except in two scenarios (Outliers = 10, no leverage and Outliers = 50, leverage = 20) where we slightly lag behind. In terms of our proposed Spatial-hard-IPOD and Spatial-soft-IPOD models, the Spatial-soft-IPOD consistently outperforms the Spatial-hard-IPOD in all situations, while the MSEs of both methods increase as the number of outliers increases (Tables 3 and  4, Figs. 3 and  4).

When *p* equals 50, the overall performance situation remains largely unchanged, with our proposed Spatial-Θ-IPOD model still exhibiting the best MSE performance among all the models. The only difference is that the MSE of Spatial-Θ-IPOD is slightly larger compared to that of *p* equals 15 (Tables 3 and  4, Figs. 3 and  4).

## Empirical study

In this section, we conducted a multi-country cross-sectional study using public data from the World Bank (https://data.worldbank.org/) among 267 countries and regions to detect outliers in life expectancy (LE) measurement for the year 2020. In order to ensure that missing values will not affect the results of our empirical study, we excluded data with missing values from some countries, resulting in a selection of 82 countries and regions. The adjacency matrix for these countries was obtained using GeoDa (Luc Anselin 1.22.0.2).

Following the variables chosen by Ranabhat (2018) [26], the dependent variable in our study is the life expectancy of each country, while the independent variables include economic growth rate, child immunization rate, out-of-pocket expenditure percentage, domestic private health expenditure percentage, and access to improved sanitation percentage.

The fitting model is

10 $${\mathbf{y}} = {\mathbf{X\beta }} + {{\varvec{\upgamma}}} + {{\varvec{\upxi}}},{{\varvec{\upxi}}} = \mu {\mathbf{W}}^{ * } {{\varvec{\upxi}}} + \epsilon,\quad \epsilon\sim {\mathcal{N}}\left( {0,\sigma^{2} {\mathbf{I}}_{82} } \right)$$

${\mathbf{W}}^{{\mathbf{*}}}$ is spatial contiguity matrix which contains the distance between each country. Because the performance of Spatial-soft-IPOD is slightly better than Spatial-hard-IPOD in our simulation, we apply our proposed Spatial-soft-IPOD to conduct this empirical study.

The results shows that the $\gamma_{10} = 11.82855$, while other $\gamma_{i} = 0$, it indicates that the 10th observation is an outlier in this situation, which is Suriname, a country in South America. The corresponding map of these countries with one outlier observation (red dot) is shown as Supplementary Fig. 1.

Accurately detecting outliers has many implications, including detecting outliers often provides valuable insights about the dataset. We furthermore conducted a thorough check of all variables for this country. Suriname's life expectancy ranks 42nd among the 82 countries, while its rankings for the remaining five indicators all fall behind 43rd. Specifically, the rankings for the other indicators are as follows: economic growth rate (81st), child immunization rate (82nd), out-of-pocket expenditure percentage (55th), domestic private health expenditure percentage (47th), and access to improved sanitation percentage (65th).

Generally, these factors all have a positive correlation with life expectancy. Under this assumption, the Suriname’s life expectancy should not rank as high as 42nd. However, the life expectancy of Suriname does not seem to align with the general trend. Therefore, it has been identified as an outlier based on these five variables.

Subsequently, we endeavored to determine the reasons behind the occurrence of this outlier. We examined other predictors related with life expectancy but not included in the study. For instance, Suriname's rankings in current health expenditure, enrollment, external health expenditure, and population growth are 39th, 22nd, 41st, and 42nd, respectively, which are higher than life expectancy ranks 42nd. Therefore, in the study, Suriname has been identified as an outlier, which may be associated with our choice of variables.

## Discussion

In this study, we proposed Spatial-Θ-IPOD for detecting spatial data outliers in SEM structures, while providing robust coefficient estimation results. We extended the IPOD method to incorporate spatial data structures, allowing for consideration of spatial error lag effects and inheriting the desirable properties of IPOD in combating masking.

In addition, due to the potential inadequacy of relying solely on raw residuals for effectively detecting outliers occurring at leverage points. Therefore, we not only examined the impact of outliers but also investigated the influence of leverage points on outlier detection, an aspect that has been rarely addressed in previous spatial outlier detection studies. Our simulation results demonstrated that the original IPOD method was not effective in detecting outliers in the presence of spatial correlation. Our masking and JD indicators outperformed several commonly used methods, both robust and non-robust, even in high-dimensional settings, with stable algorithm performance. While outlier detection was our primary objective, our model also provided stable coefficient estimation. Simulation study showed that our algorithm performed better than other models in the majority of cases, with only slight inferiority to the RoMLE model in a few instances. Furthermore, the MSE of our method slightly increased with increasing data contamination, which is consistent with general knowledge.

Accurately detecting outliers is important because it provides valuable insights about the dataset. The empirical study given is of Suriname being identified as an outlier observation in a study. The rankings of Suriname in various indicators, such as life expectancy and other variables, do not align with the general trend. This exemplifies one aspect of the significance of outlier detection, as analyzing outlier points can provide additional information. As demonstrated in this example, it indicates that the selected variables cannot fully explain all observations. When other four relevant variables are included in the model, Suriname is no longer classified as an outlier. Outliers offer valuable insights for uncovering hidden knowledge and enhancing healthcare services. Medical professionals can utilize these results to make informed predictions from extensive medical databases.

A limitation of this study is that in our simulation study, we have not considered the case of *p* > *n*. Currently, there are some issues with inadequate sample sizes in existing research, which will be the focus of our future studies. Another limitation of this study is that we tailored for cross-sectional data analysis rather than longitudinal data. The longitudinal data offers benefits such as capturing temporal trends and changes over time. We intend to extend our model to longitudinal data in future research.

## Conclusion

In conclusion, we proposed a Spatial-Θ-IPOD method that effectively detects spatial outliers in the context of SEM structure and provides robust estimates of coefficients. Our method demonstrates relative superiority even in the presence of high leverage points. The detection of outliers offers valuable insights and enhances our understanding of the data.

### Supplementary Information

**Supplementary Material 1.** 

## Data Availability

All data involved in the current empirical study were obtained from World Bank program (https://data.worldbank.org/).
