# Supplementary material for: Outlier detection in spatial error models using modified thresholding-based iterative procedure for outlier detection approach
Source: BMC Med Res Methodol. 2024 Apr 15;24:89. doi: 10.1186/s12874-024-02208-3 (PMC11323683; doi:10.1186/s12874-024-02208-3)
Supplement: Supplementary file 1 — Supplementary Material 1. [file 12874_2024_2208_MOESM1_ESM.docx]

Supplementary material

Another empirical study we conducted is about male lip cancer in Scottish districts from 1975 to 1980. The dependent variable is number of lip cancer cases diagnosed from 1975 to 1980. The independent variable are total person years at risk from 1975 to 1980 (X_1_), the fraction of the population involved in outdoor industry (agriculture, fishing, and forestry) which exposes it to sunlight (X_2_) and the expected cases of lip cancer during the from 1975 to 1980 (X_3_). The variable choice is according to Brewslow et al.[1].We includes 56 Scottish districts. The adjacency matrix *W^*^* contains the graph distances between 56 districts. Using the same method as the previous empirical study, we detected 4 outliers among 56 observations, namely Banff-Buchan, Moray, Aberdeen and Glasgow. The **of these four observations are 18.4314, 5.1367, 4.4925, and -4.7389. We checked the number of lip cancer cases diagnosed from 1975 to 1980 of these four districts are all more than 26 cases, while the mean cases of all districts is 9.5714. Apparently, the cases in these four districts are serious deviation from the overall.


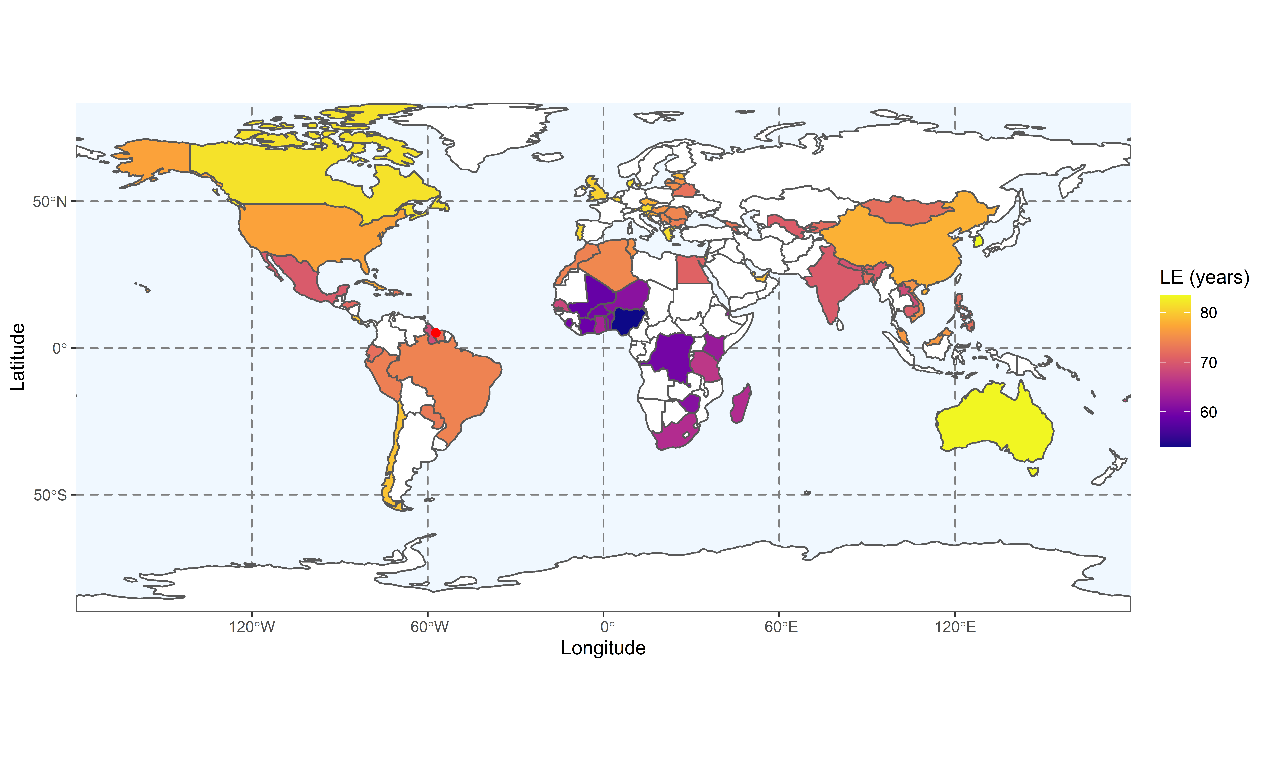


Supplementary Figure 1. The map of 82 countries with one outlier (red dot)
